# Reliability of M-wave amplitude measurement from paired stimulations across different muscle groups before and after submaximal contraction to failure

**DOI:** 10.1007/s00421-026-06259-w

**Published:** 2026-05-26

**Authors:** C. Lanfranchi, D. Neyroud, J. Rodriguez-Falces, N. Place

**Affiliations:** 1 Institute of Sport Sciences, University of Lausanne, Lausanne, Switzerland; 2 Department of Biomedical Sciences, University of Lausanne, Lausanne, Switzerland; 3 Department of Electrical and Electronical Engineering, Public University of Navarra, Pamplona, Spain; 4 Quartier UNIL-Centre, Bâtiment Synathlon, Lausanne, 1015 Switzerland

**Keywords:** Compound muscle action potential, Fatigue, Doublet, Sarcolemmal excitability

## Abstract

**Purpose:**

In exercising humans, contractility is usually investigated using paired/tetanic transcutaneous electrically-evoked contraction, while single stimulations are used to quantify sarcolemmal excitability through M-wave amplitude. Here, we aim at investigating the reliability and validity of M-wave peak-to-peak amplitude and first phase amplitude obtained from paired stimulation compared to those obtained from single stimulation (gold standard) before and after a sustained submaximal isometric contraction of the knee extensor, plantar flexor, elbow flexor and thumb adductor muscles.

**Methods:**

M waves, in response to single and paired (10 Hz and 100 Hz) stimuli, were collected in thirteen young, healthy men (25 ± 2 year) before and immediately after an isometric contraction sustained at 50% maximal voluntary force until failure. Lin’s concordance correlation coefficient was used to measure the reliability of M-wave parameters obtained from single and paired stimuli.

**Results:**

Variability in the changes in M-wave amplitude was observed following exercise depending on the muscle considered and underscores the need to analyze both phases of the M wave. This study also demonstrates that the M-wave elicited by the first stimulation in a 10-Hz (as opposed to 100-Hz) paired pulse provides an accurate and reliable estimate of sarcolemmal excitability under both resting and fatigued conditions across all investigated muscles.

**Conclusion:**

When using 10-Hz paired stimuli to assess peripheral adjustments, single stimulations may be unnecessary for evaluating sarcolemmal excitability.

**Supplementary Information:**

The online version contains supplementary material available at https://doi.org/10.1007/s00421-026-06259-w.

## Introduction

The muscle compound action potential (M-wave) amplitude is used as an index of sarcolemmal excitability (Hicks and McComas 1989), usually obtained by applying single supramaximal transcutaneous electrical stimulation over the nerve trunk or the intramuscular nerve branches. To characterize any defect in excitation-contraction coupling after exercise or between different populations, the M wave is usually quantified in parallel with the mechanical response known as the muscle twitch. As the amplitude of the twitch provides limited information on the contractile status of the muscle (Froyd et al. 2013), single stimulations are often combined with paired-/tetanic electrically-evoked contractions delivered at high and low stimulation frequencies. It was shown by Verges et al. (2009) that paired stimuli delivered at 10 and 100 Hz, which are better tolerated than tetanic contractions, can be used in the knee extensors to quantify the 10/100 Hz ratio to assess prolonged low-frequency force depression after exercise. It has also been shown that the use of paired stimuli (usually 100 Hz evoked during and after the maximal voluntary contraction (MVC)) is preferable over single shocks to quantify the maximal voluntary activation level (VAL) with the twitch interpolation technique (Duchateau 2009, Place et al. 2007). Thus, a commonly used sequence to characterize neuromuscular fatigability is to deliver a 100-Hz paired stimulus during the course of an MVC, followed by another 100-Hz paired stimulus, a 10-Hz paired stimulus and a single stimulus at rest over the nerve trunk to respectively quantify VAL/central fatigue, contractility/peripheral fatigue (100-Hz amplitude and 10/100 Hz ratio) and sarcolemmal excitability (M-wave amplitude in response to single stimulus) (Froyd et al. 2013, Lanfranchi et al. 2024, Neyroud et al. 2013). Given the potential discomfort associated with transcutaneous electrical stimulations (Cattagni et al. 2018), any strategy aiming at reducing the number of stimulations would be beneficial especially for testing frail populations. The possibility of quantifying the M-wave parameters using paired stimulations would be beneficial in such contexts. However, stimulation artifacts or overlapping M-wave responses may distort the first or second M wave of the doublet, making it difficult to be reliably quantified. Since the interval between the artifact and the M wave varies depending on anatomical parameters, as well as on the characteristics of the detection and stimulation systems, the feasibility of using a doublet to assess M-wave properties must be carefully evaluated.

While the M-wave peak-to-peak amplitude (AMPL_P−P_) has been the most widely used index for assessing sarcolemmal excitability (Hicks and McComas 1989, Cupido et al. 1996, Ducrocq and Blain 2022, Lepers et al. 2002), the first and second phases of the M wave reflect distinct properties of the muscle action potential. The first phase (AMPL_1st_) represents the propagation of the action potential along the sarcolemma, while the second phase (AMPL_2nd_) mostly results from the action potential’s extinction at the aponeurosis-tendon end (Rodriguez-Falces et al. 2022). Due to the intrinsic differences between these phases, AMPL_1st_ has been suggested as a better index for investigating sarcolemmal excitability (Lanfranchi et al. 2024, Rodriguez-Falces and Place 2017, Rodriguez-Falces and Place 2019), while AMPL_2nd_ changes would rather reflect architectural changes (Rodriguez-Falces and Place 2018). Furthermore, the M-wave amplitude measured in different muscle groups could be differently affected by a given fatiguing task. Muscle typology has been suggested to influence the fatigue-induced changes in sarcolemmal excitability due to differences in sarcolemmal membrane characteristics (Enoka et al. 1992, Everts et al. 1988, Juel 1986, Kupa et al. 1995). Therefore, to have a full picture, M-wave amplitude using single or paired stimulation needs to be assessed both at rest and in a fatigued state, in various muscle groups and using separate analysis of AMPL_1st_ and AMPL_2nd_.

Therefore, the primary aim of this study was to investigate the reliability and validity of AMPL_P−P_ and AMPL_1st_ obtained from paired stimulation compared to those obtained from single stimulation (gold standard) before and after a sustained submaximal isometric contraction of the knee extensor (KE), plantar flexor (PF), elbow flexor (EF) and thumb adductor (THU) muscles. The secondary aim was to determine whether AMPL_1st_ and AMPL_2nd_ respond differently to a sustained submaximal contraction performed to failure across multiple muscle groups.

## Materials and methods

Data presented in this study originate from a previous study (Neyroud et al. 2013). Results related to MVC force, twitch amplitude and M-wave peak-to-peak amplitude obtained from single stimulation were already presented in the original publication.

### Participants and ethical approval﻿.

Thirteen healthy men (25 ± 2 year, 177 ± 7 cm, 72 ± 3 kg) volunteered to participate in the study after having been informed of the experimental procedures and possible risks. They were all recreationally active but were not involved in any structured training program. The study protocol was approved by the Ethics Committee of the University Hospitals of Geneva (protocol 10–238) and conformed to the declaration of Helsinki. Before participating, each participant gave written informed consent.

### Experimental protocol

The experimental protocol and set-up of the present study has been described in the original publication (Neyroud et al. 2013). Briefly, participants reported to the laboratory on two separate occasions separated by at least 48 h. To account for circadian changes, the two visits were performed at the same time of the day for a given participant. One upper (EF or THU) and one lower (i.e. KE or PF) limb muscle group were tested within a single session. A 50-min resting period was given to the participant between the upper- and lower-body tasks to limit the potential influence of the first fatiguing task on the second fatiguing task that engaged a different muscle group. The order of the two sessions was randomized, and the order between tasks within a session was counterbalanced between participants.

**Fig. 1:**
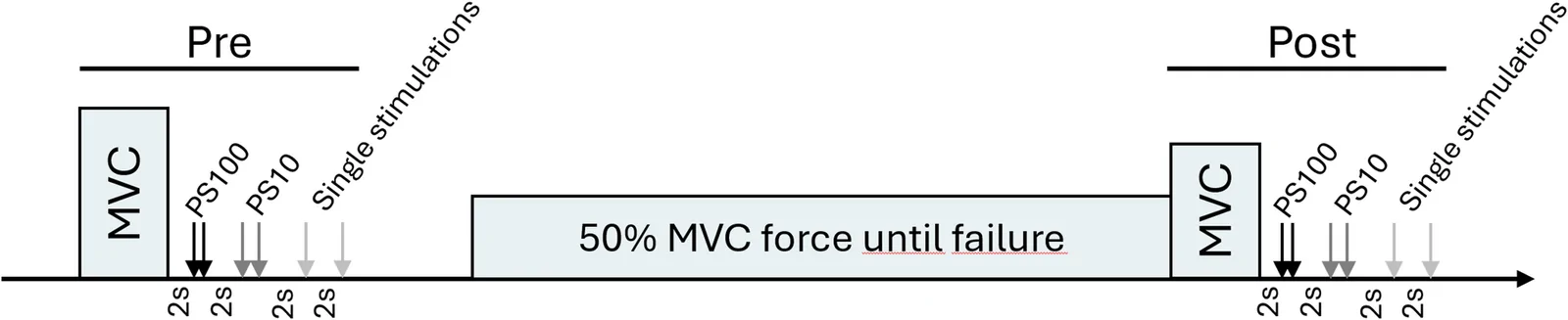
Experimental protocol scheme. MVC, maximal voluntary contraction; PS100, 100-Hz paired stimuli; PS10, 10-Hz paired stimuli. Adapted from Neyroud et al. (2013).

Each session began with a warm-up of 8 to 10 submaximal voluntary isometric contractions (4–5 s) realized between 20 and 80% of the estimated MVC force. Pre fatigue tests consisted in two to three MVCs (duration of ~ 4 s) separated by 1 min of rest. Each MVC was followed (2 s intervals) by paired electrical stimuli at 100 Hz and 10 Hz (hereafter referred to as PS100 and PS10, respectively), and then two single stimulations were delivered in a relaxed state (Pre). No more than 5% variation between the two highest MVCs was tolerated. Afterwards, participants performed a fatiguing task consisting in a sustained submaximal voluntary isometric contraction of the muscles at a force corresponding to 50% of MVC force until task failure. Live force feedback was displayed on a computer screen, and the target force was indicated with a horizontal line. Participants were asked to remain as close as possible to this target line. Task failure was defined as the inability of the participant to reach the target force for more than 3 consecutive seconds despite strong verbal encouragement (Neyroud et al. 2013). Throughout the study, the same experimenter determined when task failure occurred. At the end of the fatiguing contraction, the exact same procedure as for the pre fatigue tests was performed, but only one MVC was performed (Post, see Fig. 1).

### Data collection

A high-voltage (400 V maximum) constant-current stimulator (DS7AH; Digitimer, Hertfordshire, UK) was used to deliver electrical stimuli. For KE, the cathode (5-cm diameter, Dermatrode; American Imex, Irvine, CA) and the anode (5 × 10 cm; Compex, Ecublens, Switzerland) were placed over the femoral nerve at the femoral triangle level and on the lower part of the gluteal fold, respectively (Neyroud et al. 2012). For the PF, the cathode (1-cm diameter, Kendall Meditrace 100; Tyco, Markham, Ontario, Canada) was placed in the popliteal fossa, and the anode (5 × 10 cm, Compex) was located on the anterior surface of the knee (Place et al. 2009). For the EF, because stimulation of the brachial plexus elicited contractions in both agonist and antagonist muscles, preventing accurate twitch force measurements, we instead stimulated the biceps muscle belly directly. The cathode was positioned midway between the anterior border of the deltoid and the proximal elbow crease, and the anode was placed over the bicipital tendon to elicit evoked forces (Smith et al. 2007); both the anode and the cathode measured 5 × 5 cm (Compex, Ecublens, Switzerland). For the THU, the cathode and the anode (4-mm plug barhandle stimulator; SPES Medica, Genova, Italy) were located over the ulnar nerve anteriorly and just proximal to the wrist (Herbert and Gandevia 1999). In VL and VM muscles, nerve and muscle stimulations have been shown to evoke identical twitch and M-wave responses when stimulated at supramaximal intensities (Rodriguez-Falces et al. 2013). Thus, differences in stimulation sites should not preclude the comparison between EF and other muscles in the present study.

The intensity used for nerve stimulation was assessed at the beginning of the protocol. The intensity of single stimuli was incrementally increased, and the maximal intensity was reached when an increase in stimulation intensity did not induce a further increase in twitch force and AMPL_P−P_ of recorded muscles. This intensity was then further increased by 10% to ensure supramaximal stimulation and kept constant throughout the experiment (Neyroud et al. 2018). As adopted in previous studies, pulse width was set at 0.1 ms for the upper limbs (Smith et al. 2007) and at 1 ms for the lower limbs (Neyroud et al. 2012, Place et al. 2009). The use of long (1 ms) or short (0.2 ms) pulses at supramaximal intensity does not affect perceived discomfort or the assessment of contractile and EMG-related parameters in the knee extensors (Giroux et al. 2018). Therefore, it is unlikely that the difference in pulse duration applied to the upper and lower limbs could compromise the comparability between muscle groups.

All force signals were recorded at 1 kHz using an AD conversion system (MP150; BIOPAC, Goleta, CA). The force developed by the KE was recorded using an isometric ergometer consisting of a custom-built chair equipped with a strain gauge (STS 2500 N, sensitivity 2.0005 mV/V and 0.0017 V/N; SWJ, Shenzhen, China). The strain gauge was attached to the chair on one end and securely strapped to the ankle with a custom-made mold. Participants were seated with a knee angle of 90° and a trunk-thigh angle of 100° (180° = full extension). Extraneous movements of the upper body were limited by two crossover shoulder harnesses and a belt across the lower abdomen. Force developed by the PF was recorded using an isometric ergometer consisting of a custom-built pedal equipped with a strain gauge (S2 1000 N, sensitivity 2 mV/V and 0.0043 V/N; HBM, Darmstadt, Germany). The foot was strapped to the pedal at the ankle as well as at the metatarsi level. Ankle and knee joints were fixed at 90°, and the trunk-thigh angle was 100°. To limit the contribution of muscle groups other than PF and to optimize force recordings, the upper leg was clamped down to the chair just proximal to the knee. Force developed by the EF was recorded using a custom-built ergometer equipped with a strain gauge (SAS 2000 N, sensitivity 1.998 mV/V and 0.0014 V/N; SWJ) and custom-made molds to support the arm and the forearm. Participants were seated with the arm flexed at 90° and the forearm vertical and supinated with the participant’s hand holding an adjustable handle connected to the strain gauge. The shoulder-trunk angle was 90° in the sagittal axis. Force developed by THU was recorded using an isometric ergometer consisting of a mold for the front arm and equipped with a strain gauge (Z8 500 N, sensitivity 2 mV/V and 0.0083 V/N; HBM) attached to a thumb support. Straps were used to minimize hand and arm contribution to force production. The forearm was at 45° from full supination toward full pronation. The thumb’s proximal phalanx rested on a support connected to the strain gauge. Elbow and shoulder were positioned at 90° in the sagittal axis, and the thumb’s adduction angle was determined as the angle allowing optimal force production for each participant during the familiarization session. All force signals were amplified with a gain of 1000.

Electromyographic (EMG) activity from the KE (*vastus lateralis* [VL], *vastus medialis* [VM], *and rectus femoris* [RF]), PF (*soleus* [SOL], *gastrocnemius lateralis* [GL], *and gastrocnemius medialis* [GM]), EF (*brachioradialis* [BRA]*)* and THU (*adductor pollicis* [ADD],) was recorded. For EF, only the *brachioradialis* muscle was assessed because of the large stimulation artifact in biceps brachii due to the position of the stimulation electrodes surrounding the EMG electrodes.

For all muscles, EMG activity was recorded with pairs of silver chloride (Ag/AgCl) circular surface electrodes (Kendall Meditrace 100) positioned according to SENIAM recommendations (Hermens et al. 2000). Specifically, for the SOL, the electrodes were placed distally at a location corresponding to 2/3 of the distance between the medial condyle of the femur and the medial malleolus. For GL and GM, the electrodes were positioned lengthwise over the middle of the muscle belly (Place et al. 2009). The reference electrodes were placed over nearby bony protuberances. Low resistance between the two electrodes (< 10 kΩ) was consistently obtained by cleaning, shaving and lightly abrading the skin. EMG signals were amplified with a gain of 1000, filtered with a bandwidth frequency between 10 Hz and 500 Hz, digitized at a sampling frequency of 2 kHz and recorded by the AD conversion system. Isometric force and EMG data were stored and analyzed offline with a commercially available software (AcqKnowledge version 4.2, BIOPAC Systems).

### Data analysis

The MVC force considered as peak force attained during the MVC and the twitch amplitude in response to single stimulation were measured before and after the fatiguing contraction.

For all muscles, AMPL_1st_, AMPL_P−P_ were measured from the EMG response (see Fig. 3) associated with the second stimulation of PS100 (M100_2nd_), both stimulations of PS10 (M10_1st_ and M10_2nd_) and the twitch stimulation (M_single_). In addition, AMPL_2nd_ was also quantified for M_single_. For the THU, the AMPL_1st_ evoked in response to the first stimulation of PS100 (M100_1st_) was also considered as the smaller amplitude of the artifact rendered possible proper analysis without contamination from the second stimulation.

### Statistical analysis

Due to poor EMG signal quality or superposition of M-waves from adjacent stimuli (as observed for M100), which prevented reliable measurement of M-wave parameters, some participants were excluded from specific muscle EMG analyses. The sample size for each muscle and corresponding M-wave measurement is reported in Table S1.

Lin’s concordance correlation coefficient (CCC) was used to measure precision and accuracy of M100_1st_, M100_2nd_, M10_1st_ and M10_2nd_ in comparison to M_single_. The absolute and relative reliability of M-wave parameters associated with consecutive M_single_ (gold standard) were also computed. CCC was defined as follows:

$$\:CCC=\frac{2{\sigma\:}_{12}}{({\mu\:}_{1}-{\mu\:}_{2}{)}^{2}+{\sigma\:}_{1}^{2}+{\sigma\:}_{2}^{2}}$$

With $\:{\sigma\:}_{12}$ being the covariance between the two variables, $\:{\sigma\:}_{1}\:\mathrm{o}\mathrm{r}\:{\sigma\:}_{2}$ the variance and $\:{\mu\:}_{1}\:\mathrm{o}\mathrm{r}\:{\mu\:}_{2}$ the average of both variables. This coefficient measures both the precision and accuracy of the relationship between two variables (Lin 1989). CCC values were interpreted as > 0.99 = almost perfect, 0.95–0.99 = substantial, 0.90–0.95 = moderate and < 0.90 = poor (McBride 2005). CCC was calculated on M waves before and after the fatiguing contraction for all muscles. In addition, CCC was also calculated on the Pre to Post changes (Δ Post-Pre) for all M waves.

Normality of data was tested using a Shapiro-Wilk test. Differences between Pre and Post for MVC force, AMPL_P−P_, AMPL_1st_ and AMPL_2nd_ were statistically analysed using Student paired t-test or Wilcoxon signed-rank test when normality assumption was not validated. The same statistical analysis was performed to compare Pre to Post changes between AMPL_1st_ and AMPL_2nd_. All statistical analyses were performed using the *Jamovi software* (version 2.3, Sydney, Australia). The α-level for statistical significance was set at *p* ≤ 0.05. Data are presented as mean change percentage ± SD in the text.

## Results

### Change in the amplitude of M-wave phases following the fatiguing contraction

The sustained submaximal contraction to failure induced a significant decrease in MVC force for all muscle groups (KE: −34.0 ± 15.4%, PF: −30.2 ± 11.0%, EF: −39.8 ± 11.7%, THU: −36.8 ± 12.8%, *p* < 0.001). The following M wave parameters were obtained from M_single_. For the knee extensors, VM AMPL_1st_ was significantly reduced (−7.8 ± 10.7%, *p* = 0.046, Table 2), whereas AMPL_P−P_ (−7.4 ± 12.1%, *p* = 0.064) and AMPL_2nd_ (−7.9 ± 19.6%, *p* = 0.105) remained unchanged. In contrast, no differences were observed in any of the VL and RF M-wave parameters. For the plantar flexors, the sustained contraction induced a decrease in SOL AMPL_P−P_ (−29.2 ± 21.3%, *p* < 0.001), AMPL_1st_ (−23.6 ± 20.4%, *p* = 0.001) and AMPL_2nd_ (−34.5 ± 25.4%, *p* < 0.001, Table 2). GM AMPL_P−P_ and AMPL_2nd_ were increased (+ 28.0 ± 28.9%, *p* < 0.001 and + 44.7 ± 51.8%, *p* = 0.011, respectively, Table 2), but not AMPL_1st_ (+ 8.0 ± 22.3%, *p* = 0.126). No change occurred for GL. Similarly, BRA M-wave amplitudes were not significantly changed in response to the sustained contraction. Finally, the ADD AMPL_P−P_ (−28.8 ± 17.7%, *p* = 0.004),  AMPL_1st_ (−30.4 ± 16.8%, *p* = 0.002) and AMPL_2nd_ (−31.2 ± 17.5%, *p* = 0.027) were significantly reduced.

**Table 1:** Knee extensor (KE), plantar flexor (PF), elbow flexor (EF) and thumb adductor (THU) M-wave peak-to-peak, 1^st^ phase and 2^nd^ phase amplitudes recorded before (Pre) and after (Post) a sustained submaximal contraction sustained to failure

		Peak-to-peak amplitude (mV)		1^st^ phase amplitude (mV)			2^nd^ phase amplitude (mV)		
		Pre	Post	Pre		Post	Pre		Post
KE	VL	12.3 ± 4.1	12.7 ± 4.4	6.2 ± 2.6		6.3 ± 2.4	6.1 ± 2.1		6.3 ± 2.4
	VM	13.1 ± 4.8	11.9 ± 4.3	7.7 ± 2.8		7.1 ± 2.8*	5.0 ± 3.0		4.2 ± 2.2
	RF	8.0 ± 1.6	7.6 ± 1.7	5.6 ± 1.2		5.5 ± 1.4	2.3 ± 0.8		2.1 ± 0.9
PF	SOL	12.5 ± 3.5	8.7 ± 3.4*	6.4 ± 2.3		4.7 ± 2.0*	6.1 ± 1.7		4.0 ± 1.8*
	GL	12.3 ± 8.1	12.3 ± 8.5	5.3 ± 2.7		4.7 ± 2.7	5.8 ± 3.9		6.4 ± 4.0
	GM	9.3 ± 5.0	11.0 ± 4.7*	4.1 ± 2.6		4.4 ± 2.8	5.3 ± 2.9		6.8 ± 2.6*
EF	BRA	3.9 ± 2.4	3.6 ± 2.6	2.3 ± 1.2		2.2 ± 1.6	1.6 ± 1.3		1.4 ± 1.2
THU	ADD	1.7 ± 2.1	1.2 ± 1.5*	0.9 ± 1.1		0.6 ± 0.7*	0.8 ± 1.1		0.6 ± 0.9*

M-wave parameters were measured from M_single_, Values are means ± SD

*VL* Vastus Lateralis, *VM* Vastus Medialis, *RF* Rectus Femoris, *Sol* Soleus, *GL* Gastrocnemius Lateralis, *GM* Gastrocnemius Medialis, *BRA* Brachioradialis, *ADD* Adductor pollicis

*Different from Pre at *p* < 0.05

**Fig. 2:**
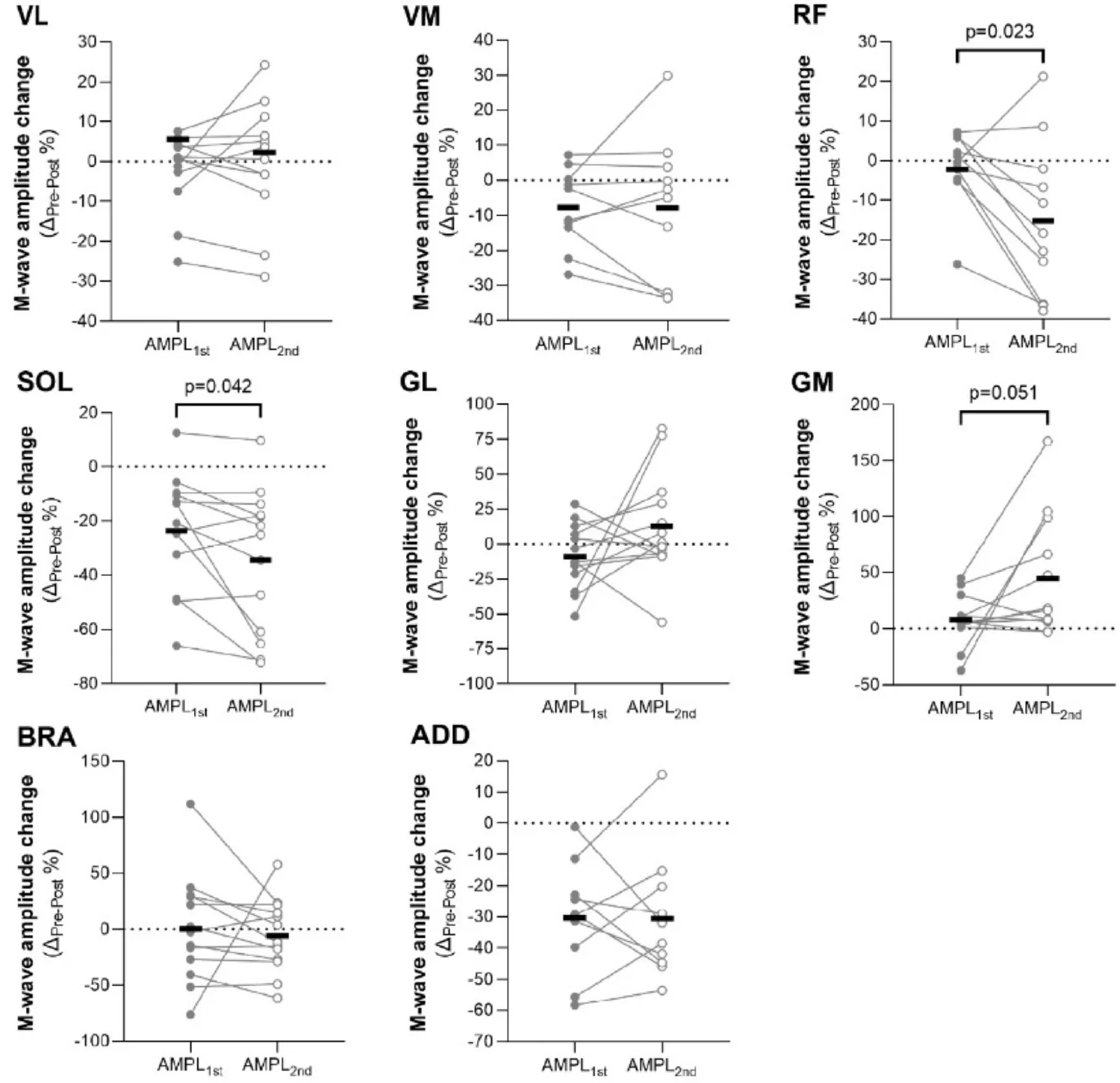
Changes in M-wave 1^st^ (AMPL_1st_) and 2^nd^ phase amplitude (AMPL_2nd_) in various muscle groups between before (Pre) and after (Post) a sustained submaximal voluntary contraction until failure. M waves parameters were measured from M_single_. Dots linked by lines represent individual participants, and black thick lines represent the mean of 1 st and 2nd phase amplitudes. *VL* Vastus Lateralis, *VM* Vastus Medialis, *RF* Rectus Femoris, *Sol* Soleus, *GL* Gastrocnemius Lateralis, *GM* Gastrocnemius Medialis, *BRA* Brachioradialis, *ADD* Adductor pollicis

When comparing AMPL_1st_ and AMPL_2nd_, we observed differences in the Pre- to Post-task changes across various muscles (Fig. 2). Specifically, for RF and SOL, the fatiguing task led to a greater reduction in AMPL_2nd_ vs. AMPL_1st_ (−15.2 ± 18.5% vs. −2.2 ± 8.6%, *p* = 0.023 and − 34.5 ± 25.4% vs. −23.6 ± 20.4%, *p* = 0.042 respectively), while for GM it tended to lead to a greater increase in AMPL_2nd_ than AMPL_1st_ (+44.7 ± 51.8% vs. +7.8 ± 22.3%, *p* = 0.051).

### Reliability of AMPL_P−P_ and AMPL_1st_ obtained from paired stimuli

**Fig. 3:**
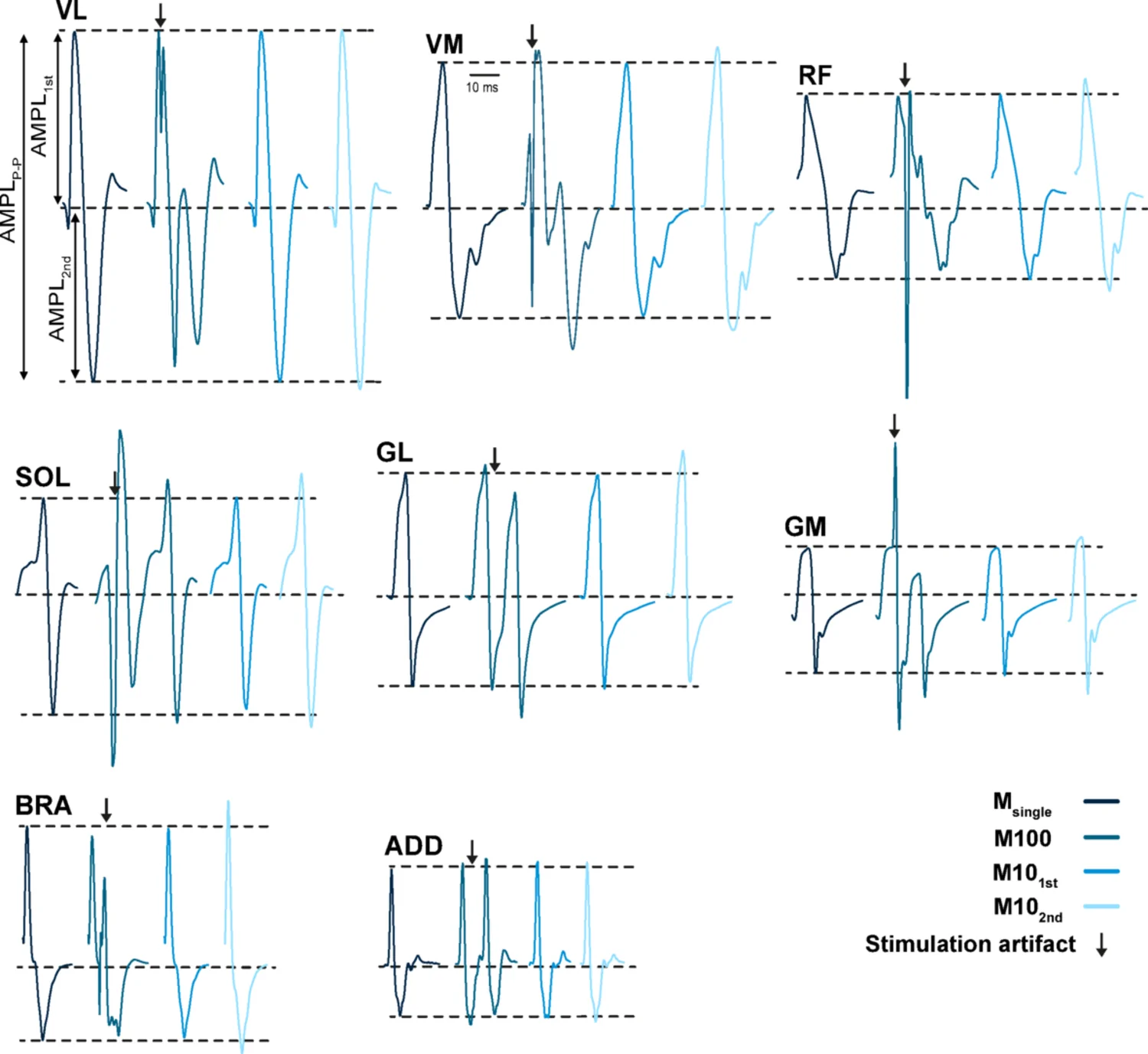
Original recordings of M waves obtained in response to a single stimulation (M_single_), the first and second stimulation of a 100-Hz paired stimuli (M100), the first and the second stimulation of a 10-Hz paired stimuli (M10_1st_ and M10_2nd_ respectively) in various muscle groups. *VL* Vastus Lateralis, *VM* Vastus Medialis, *RF* Rectus Femoris, *Sol* Soleus, *GL* Gastrocnemius Lateralis, *GM* Gastrocnemius Medialis, *BRA* Brachioradialis, *ADD* Adductor pollicis

**Fig. 4:**
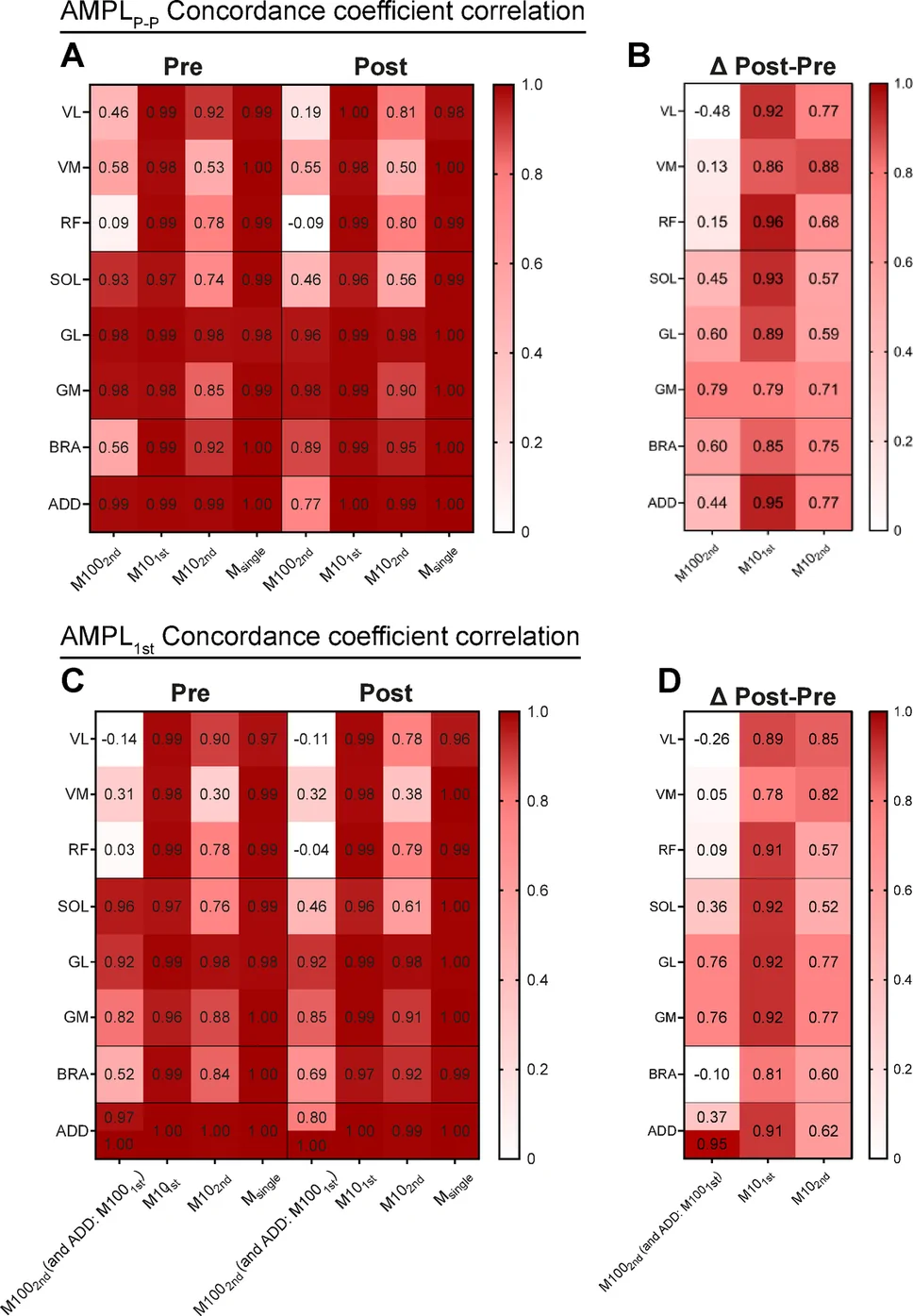
**A** and **B**, heatmaps of concordance coefficient correlation (CCC) between M-wave peak-to-peak amplitudes (AMPL_P−P_) obtained (**A**) before (Pre) and after (Post) a sustained submaximal voluntary contraction until failure or between (**B**) AMPL_P−P_ Pre to Post changes values (Δ Post-Pre). **C** and **D**, idem as in **A** and **B** but for 1^st^ phase amplitudes (AMPL_1st_). M waves obtained in response to a single stimulation were compared to M waves obtained in response to the first (only for ADD muscle on AMPL_1st_) and second stimulation of a 100-Hz paired stimuli (M100_1st_ and M100_2nd_ respectively, M100_2nd_ values are above M100_1st_ values for ADD), the first and second stimulation of a 10-Hz paired stimuli (M10_1st_ and M10_2nd_ respectively) and another single stimulation (M_single_). *VL* Vastus Lateralis, *VM* Vastus Medialis, *RF* Rectus Femoris, *Sol* Soleus, *GL* Gastrocnemius Lateralis, *GM* Gastrocnemius Medialis, *BRA* Brachioradialis, *ADD* Adductor pollicis

M_single_ reproductibility was very high in all muscles for both AMPL_P−P_ and AMPL_1st_ (Fig. 4). The best CCC when comparing M_single_ with M waves from paired stimuli was obtained with M10_1st_ for all muscle groups (Fig. 3A and C). In addition, the CCC calculated between M10_1st_ and M_single_ were close to those calculated between two M_single_ (Fig. 4A and C). M10_2nd_ was less reliable than M10_1st_ for all muscle groups, but still performed well for GL and ADD muscles (Fig. 4A and C). Amplitude of M10_2nd_ was significantly higher than amplitude of M10_1st_ in RF, SOL and GM muscles (Table 2). Overall, M100_2nd_ was the least reliable M-wave parameter for KE and EF, while M100_1st_ could not be quantified in most muscles due to the contamination of the second stimulation artifact (Fig. 3). However, in ADD muscle, AMPL_1st_ of M100_1st_ could be measured thanks to the very small stimulation artifact, and displayed a very high concordance coefficient (Fig. 4C). CCC calculated from Pre or Post fatigue M waves were close to each other regardless of the muscle (Fig. 4A and C). The same was observed for CCC calculated from either AMPL_P−P_ or AMPL_1st_ (Fig. 4A and C). Finally, to check the reliability of the various M waves changes over time, we also calculated the CCC on Δ Post-Pre (Fig. 4B and D) between M_single_ and other M waves. Overall it confirmed the high reliability of M10_1st_ as compared to M10_2nd_ and M100_2nd_ for both the AMPL_P−P_ (Fig. 4B) and AMPL_1st_ (Fig. 4D).

**Table 2:** M-wave peak-to-peak and 1^st^ phase amplitudes of the first (M10_1st_) and second (M10_2nd_) M wave of a 10-Hz paired stimuli from knee extensors (KE), plantar flexors (PF), elbow flexors (EF) and thumb adductors (THU) before the sustained submaximal contraction until failure.

		Peak-to-peak amplitude (mV)		Variation, %	1^st^ phase amplitude (mV)			Variation, %
		M10_1st_	M10_2nd_		M10_1st_		M10_2nd_	
KE	VL	12.3 ± 4.1	11.9 ± 4.3	−1.2 ± 24.7	6.2 ± 2.3		5.8 ± 2.3	−3.6 ± 30.3
	VM	13.4 ± 5.2	15.5 ± 5.4	+ 8.4 ± 19.7	7.5 ± 2.7		9.2 ± 3.7	+ 9.9 ± 20.6
	RF	7.5 ± 1.8	8.2 ± 2	+ 11.0 ± 17.0	5.4 ± 1.3		5.9 ± 1.3*	+ 12.4 ± 14.8
PF	SOL	12.6 ± 3.7	14.9 ± 3.7*	+ 20.7 ± 14.6	6.6 ± 2.4		7.9 ± 2.5*	+ 23.8 ± 17.5
	GL	12.3 ± 8.8	13.0 ± 8.8	+ 7.0 ± 14.1	5.2 ± 2.7		5.7 ± 3.0	+ 12.1 ± 24.0
	GM	9.1 ± 5.0	11.3 ± 5.2*	+ 30.6 ± 28.9	4.1 ± 2.7		5.2 ± 2.9*	+ 33.3 ± 38.0
EF	BRA	3.8 ± 2.2	4.4 ± 3.1	+ 11.1 ± 25.0	2.2 ± 1.1		2.6 ± 1.8	+ 9.0 ± 34.6
THU	ADD	1.7 ± 2.1	1.8 ± 2.3	+ 0.5 ± 15.0	0.9 ± 1.1		0.9 ± 1.1	−2.1 ± 13.3

Values are means ± SD

*VL* Vastus Lateralis, *VM* Vastus Medialis, *RF* Rectus Femoris, *Sol* Soleus, *GL* Gastrocnemius Lateralis, *GM* Gastrocnemius Medialis, *BRA* Brachioradialis, *ADD* Adductor pollicis

*Different from M10_1st_ at *P* < 0.05

## Discussion

Two main findings can be drawn from the present study. First, the first phase of the M wave (AMPL_1st_, which reflects the propagation of the action potential along the sarcolemma) and the second phase of the M wave (AMPL_2nd_, which reflects its extinction at the aponeurosis-tendon end) showed distinct behaviours in response to a sustained submaximal fatiguing contraction depending on the muscle considered (PF, KE, EF and THU). Second, sarcolemmal excitability can be assessed using the M wave recorded in response to the first stimulation of a 10-Hz paired stimuli, as both AMPL_1st_ and AMPL_P−P_ associated with these paired-stimulations were found to be as reliable as the AMPL_1st_ and AMPL_P−P_ recorded in response to a single stimulation, even after a fatiguing task.

### Change in M-wave parameters from M_single_ following the fatiguing contraction

While maximal force generating capacity was depressed after the sustained contraction in all muscle groups, not all muscles exhibited alterations in sarcolemmal excitability, as reflected by AMPL_P−P_ or AMPL_1st_. This variability has already been observed in several studies using AMPL_P−P_ (Rodriguez-Falces and Place 2018), highlighting inter-muscle differences in sarcolemmal alterations in response to voluntary contractions (Place et al. 2010). Notably, our findings reveal that sarcolemmal alterations induced by submaximal fatiguing contractions can differ among muscles of a given muscle group. This is exemplified by the opposite changes observed in SOL and GM AMPL_P−P_, with SOL M waves showing a decreased amplitude, while GM M waves showed an increased amplitude. Interestingly, these changes in AMPL_P−P_ were largely driven by changes in AMPL_2nd_. Possible explanation for the difference between these two muscles may be variations in pennation angle and in the relative position of the surface electrode with respect to the muscle fiber endings. It is possible that in the present study the fibers were pennated in the GM muscle but ran parallel to the skin surface in the soleus muscle. Such geometrical and recording factors may differentially affect the summation of end-of-fiber components at the electrode across muscles (Rodriguez-Falces et al. 2022, Botter and Vieira 2017). In future research it would be interesting to investigate whether and how the direction of fibers relative to the skin (parallel or pennate) affects the changes in AMPL_1st_ and AMPL_2nd_ of an M-wave induced by a fatiguing contraction. AMPL_2nd_ was also largely decreased in ADD, which may be attributed to the short distance between the electrical generators (muscle fibers) and the recording electrodes (Dimitrova and Dimitrov 2002), as well as to the greater overlap between propagating and end-of-fiber components due to the small size of hand muscles (Rodriguez-Falces and Place 2014).

The interpretation of M-wave amplitude changes has been challenged in recent years. It has been suggested that the previously established consensus, which proposed that a decrease in AMPL_P−P_ indicates a decrease in sarcolemmal excitability (Boyas and Guével 2011), may be incorrect (Rodriguez-Falces and Place 2017). Based on results obtained in knee extensors during sustained and intermittent MVCs, Rodriguez-Falces & Place proposed that sarcolemmal excitability impairment might rather cause a potentiation of AMPL_1st_ (Rodriguez-Falces and Place 2017, Rodriguez-Falces and Place 2019, Rodriguez-Falces and Place 2018). In the present study, while the GM AMPL_P−P_ was increased following the contraction, it seemed to be primarily driven by an increase in AMPL_2nd_, likely reflecting architectural changes rather than changes in sarcolemmal excitability (Rodriguez-Falces and Place 2018). Conversely, both AMPL_1st_ and AMPL_2nd_ decreased in SOL muscle. This reduction in AMPL_1st_ likely reflects altered sarcolemmal excitability, with a decreased K⁺ gradient between the intracellular and extracellular compartments being a potential mechanism (Cairns and Lindinger 2008). Regarding VM muscle, while both AMPL_1st_ and AMPL_P−P_ decreased by around 7–8%, only AMPL_1st_ reached significance, suggesting a better sensitivity of this parameter to reflect sarcolemmal excitability. This pattern also suggests that the study may have been underpowered to detect differences consistently across all parameters, and that inclusion of additional participants might have resulted in statistically significant findings for more outcomes. Therefore, this limitation should be considered when interpreting the results.

Altogether, this suggests that alterations of sarcolemmal excitability and muscle/fiber architecture in response to a fatiguing contraction may affect the amplitudes of M wave in different directions, depending on the muscle investigated and the contraction paradigm used to induce fatigue (i.e. voluntary vs. electrically-evoked contraction). These differences may arise from the intrinsic specificity of the muscles in the considered task. It is for instance known that at a knee angle of 90°, SOL muscle is the major force contributor (Cresswell et al. 1995, Kawakami et al. 2006, Sale et al. 1982). Additionally, the GM may be more fatigable than SOL (Neyroud et al. 2013, Ochs et al. 1977) because of their distinct muscle fiber type distribution – predominantly type I in the SOL (Johnson et al. 1973).

In the present study, we also highlighted that for the same muscle, AMPL_1st_ and AMPL_2nd_ can be affected to a different extent in terms of amplitude, highlighting the distinct electrophysiological origin of the two M-wave phases. As highlighted by our team in previous studies, assessing sarcolemmal excitability using AMPL_P−P_, which inherently includes both phases, may lead to a misinterpretation of M-wave amplitude changes (Rodriguez-Falces and Place 2017, Rodriguez-Falces and Place 2019, Rodriguez-Falces et al. 2023). For instance, during a 1-s tetanic stimulation of the knee extensors and elbow flexors, Rodriguez-Falces et al. (2023) observed increases in AMPL_P−P_ of around 15–20% in the *vastus lateralis* and *biceps brachii*, while AMPL_1st_ decreased by 10–15%. Accordingly, the increase in AMPL_P−P_ was driven by an important increase in AMPL_2nd_ by 40–50%. In the present study, while we do not report opposite changes between AMPL_1st_ and AMPL_2nd_, the magnitude of change was greater for AMPL_2nd_ than for AMPL_1st_ for RF, SOL and GM, further highlighting the importance of a separate analysis of both phases.

While many studies investigated M wave changes in response to fatiguing tasks have used AMPL_P−P_ as an index of sarcolemmal excitability (Hicks and McComas 1989, Cupido et al. 1996, Ducrocq and Blain 2022, Lepers et al. 2002), the present study and others from our group have repeatedly shown that both phases of the M wave can be differently affected (Lanfranchi et al. 2024, Rodriguez-Falces and Place 2017, Rodriguez-Falces and Place 2019). This is explained by the fact that both phases reflect the two components of the extracellular potentials: the first phase corresponds to action potential propagation along the muscle fibers, whereas the second arises from action potential extinction at the myotendinous junction (Rodriguez-Falces et al. 2022). Because these phases represent different physiological processes and are influenced by multiple factors (e.g., ion concentration, muscle shortening, muscle bulging, fiber pennation, etc.) it is not surprising that they can be affected differently. This is confirmed in the present study by the distinct behavior of both phases within and between muscles. 

### Accuracy and reliability of M-waves AMPL_P−P_ and AMPL_1st_ obtained from paired stimulations

In the subsequent set of analysis aiming at investigating the accuracy and reliability of measuring AMPL_P−P_ and AMPL_1st_ in response to paired stimulations, we observed that the M10_1st_ had a very high CCC, very similar to what has been obtained with M_single_. In contrast, M10_2nd_ performed poorly as compared to M10_1st_, except for ADD and GL muscles. This may be, at least in part, due to the increased amplitude of M10_2nd_ as compared to M10_1st_ observed in almost all muscles studied. This increased amplitude of the M wave associated with the second stimulation of the 10-Hz paired stimulation may result from changes in muscle geometry induced by the first stimulation of the 10-Hz paired stimulation as it is known that changes in muscle geometry can affect M-wave amplitude (Rodriguez-Falces et al. 2022). This result therefore suggests that if paired stimulations are to be used to assess M-wave parameters, only the M wave associated with the first stimulation of the paired stimulation should be used to avoid potential muscle geometrical changes. This conclusion is strengthened with the higher CCC values obtained for M10_1st_ as compared to M10_2nd_ or M100_2nd_ when considering exercise-induced changes in M-wave amplitude.

This study further showed that while using the M wave associated with the first stimulation of a 10-Hz paired stimulation is in agreement with the conventional method of quantifying it from a single stimulus, using the M wave associated with the first stimulation of a 100-Hz paired stimulation proved difficult due to the presence of the second stimulation artifact, which prevented the measurement of M100_1st_ in all muscles except the ADD muscle. The proximity between the EMG electrodes and the site of stimulation for the ADD muscle resulted in a short latency between the stimulation artifact and the M wave (~ 5 ms), which led the second artifact to appear around the end of M100_1st_ second phase, allowing reliable measurement of AMPL_1st_ from M100_1st_ in the ADD muscle. In agreement with what we observed for the 10-Hz paired stimulation, assessing M wave associated with the second stimulation of a 100-Hz paired stimulation also led to inaccurate measurements. Indeed, and unsurprisingly due to the high stimulation frequency, the M100_2nd_ was the M wave performing the worst in terms of CCC against M_single_.

## Conclusion

Overall, this study highlights the variability in the changes in M-wave amplitude following a sustained submaximal contraction and underscores the need to differentiate between AMPL_1st_ and AMPL_2nd_. Indeed, we observed that a sustained submaximal contraction can lead to different direction of changes in AMPL_1st_ and AMPL_2nd_ depending on the muscle considered. In addition, AMPL_1st_ and AMPL_2nd_ can be differently affected within the same muscle. Therefore, relying solely on the commonly measured AMPL_P−P_ is insufficient, as any observed alterations—or lack thereof—only reflect the combined effect of AMPL_1st_ and AMPL_2nd_. Instead, future research using M-wave parameters to assess changes in sarcolemmal excitability should report phase amplitudes quantification separately.

This study also demonstrates that the M-wave elicited by the first stimulation in a 10-Hz paired pulse can serve as a surrogate to the M-wave elicited by a single pulse in both resting and fatigued conditions across all investigated muscles. In contrast, M10_2nd_, M100_1st_, and M100_2nd_ were less effective compared to M10_1st_.

This indicate that if 10-Hz and 100-Hz paired stimuli are used to quantify peripheral adjustments and that parameters such as contraction time or relaxation rate usually derived from single stimulation are not measured, the single stimulations could be avoided by considering M10_1st_ for the investigation of sarcolemmal excitability. This would not compromise excitation–contraction coupling assessment, as the mechanical response to 100-Hz paired pulse has been advocated to characterize contractile impairment after exercise (Place et al. 2007).

## Supplementary Information

Below is the link to the electronic supplementary material.

Supplementary Material 1

## References

[CR1] Botter A, Vieira TM (2017) Optimization of surface electrodes location for H-reflex recordings in soleus muscle. J Electromyogr Kinesiol 34:14–23. 10.1016/j.jelekin.2017.03.003 28342367

[CR2] Boyas S, Guével A (2011) Neuromuscular fatigue in healthy muscle: underlying factors and adaptation mechanisms. Ann Phys Rehabil Med 54(2):2. 10.1016/j.rehab.2011.01.001 21376692

[CR3] Cairns SP, Lindinger MI (2008) Do multiple ionic interactions contribute to skeletal muscle fatigue? J Physiol 586(17):4039–4054. 10.1113/jphysiol.2008.155424 PMC2652190 18591187

[CR4] Cattagni T, Merlet AN, Cornu C, Jubeau M (2018) H-reflex and M-wave recordings: effect of pressure application to the stimulation electrode on the assessment of evoked potentials and subject’s discomfort. Clin Physiol Funct Imaging 38(3):416–424. 10.1111/cpf.12431 28444940

[CR5] Cresswell AG, Löscher WN, Thorstensson A (1995) Influence of gastrocnemius muscle length on triceps surae torque development and electromyographic activity in man. Exp Brain Res 105(2):2. 10.1007/BF00240964 7498381

[CR6] Cupido CM, Galea V, McComas AJ (1996) Potentiation and depression of the M wave in human biceps brachii. J Physiol 491(Pt 2):541–550 PubMed PMID: 8866877; PubMed Central PMCID: PMC1158748 10.1113/jphysiol.1996.sp021238 PMC1158748 8866877

[CR7] Dimitrova NA, Dimitrov GV (2002) Amplitude-related characteristics of motor unit and M-wave potentials during fatigue. A simulation study using literature data on intracellular potential changes found in vitro. J Electromyogr Kinesiol 12(5):5. 10.1016/S1050-6411(02)00046-9 12223166

[CR8] Duchateau J (2009) Stimulation conditions can improve the validity of the interpolated twitch technique. J Appl Physiol 107(1):361–368. 10.1152/japplphysiol.00362.2009 19670474

[CR9] Ducrocq GP, Blain GM (2022) Relationship between neuromuscular fatigue, muscle activation and the work done above the critical power during severe-intensity exercise. Exp Physiol 107(4):312–325. 10.1113/EP090043 35137992

[CR10] Enoka RM, Trayanova N, Laouris Y, Bevan L, Reinking RM, Stuart DG (1992) Fatigue-related changes in motor unit action potentials of adult cats. Muscle Nerve 15(2):138–150. 10.1002/mus.880150204 1549136

[CR11] Everts ME, Retterstøl K, Clausen T (1988) Effects of adrenaline on excitation-induced stimulation of the sodium-potassium pump in rat skeletal muscle. Acta Physiol Scand 134(2):189–198. 10.1111/j.1748-1716.1988.tb08479.x 2852445

[CR12] Froyd C, Millet GY, Noakes TD (2013) The development of peripheral fatigue and short-term recovery during self-paced high-intensity exercise. J Physiol 591(5):1339–46. 10.1113/jphysiol.2012.245316 PMC3607875 23230235

[CR13] Giroux C, Roduit B, Rodriguez-Falces J, Duchateau J, Maffiuletti NA, Place N (2018) Short vs. long pulses for testing knee extensor neuromuscular properties: does it matter? Eur J Appl Physiol 118(2):361–369. 10.1007/s00421-017-3778-7 29218407

[CR14] Herbert RD, Gandevia SC (1999) Twitch Interpolation in Human Muscles: Mechanisms and Implications for Measurement of Voluntary Activation. J Neurophysiol 82(5):2271–2283. 10.1152/jn.1999.82.5.2271 10561405

[CR15] Hermens HJ, Freriks B, Disselhorst-Klug C, Rau G (2000) Development of recommendations for SEMG sensors and sensor placement procedures. J Electromyogr Kinesiol 10(5):5. 10.1016/S1050-6411(00)00027-4 11018445

[CR16] Hicks A, McComas AJ (1989) Increased sodium pump activity following repetitive stimulation of rat soleus muscles. J Physiol 414(1):1 10.1113/jphysiol.1989.sp017691 PMC1189145 2558169

[CR17] Johnson MA, Polgar J, Weightman D, Appleton D (1973) Data on the distribution of fibre types in thirty-six human muscles. An autopsy study. J Neurol Sci 18(1):111–29. 10.1016/0022-510x(73)90023-3 4120482

[CR18] Juel C (1986) Potassium and sodium shifts during in vitro isometric muscle contraction, and the time course of the ion-gradient recovery. Pflugers Arch 406(5):458–463. 10.1007/BF00583367. (**PubMed PMID: 3714446**) 3714446

[CR19] Kawakami Y, Abe T, Kanehisa H, Fukunaga T (2006) Human skeletal muscle size and architecture: variability and interdependence. Am J Hum Biol 18(6):6. 10.1002/ajhb.20561 17039483

[CR20] Kupa EJ, Roy SH, Kandarian SC, De Luca CJ (1995) Effects of muscle fiber type and size on EMG median frequency and conduction velocity. J Appl Physiol Bethesda Md 1985 79(1):23–32. 10.1152/jappl.1995.79.1.23. (**PubMed PMID: 7559225**) 7559225

[CR21] Lanfranchi C, Willis SJ, Laramée L, Conde Alonso S, Pialoux V, Kayser B et al (2024) Repeated sprint training in hypoxia induces specific skeletal muscle adaptations through S100A protein signaling. FASEB J 38(8):e23615. 10.1096/fj.202302084RR 38651657

[CR22] Lanfranchi C, Rodriguez-Falces J, Place N (2024) The first and second phases of the muscle compound action potential in the thumb are differently affected by electrical stimulation trains. J Appl Physiol 136(5):1122–1128. 10.1152/japplphysiol.00861.2023 38511213

[CR23] Lepers R, Maffiuletti NA, Rochette L, Brugniaux J, Millet GY (2002) Neuromuscular fatigue during a long-duration cycling exercise. J Appl Physiol 92(4):4. 10.1152/japplphysiol.00880.2001 11896014

[CR24] Lin LIK (1989) A concordance correlation coefficient to evaluate reproducibility. Biometrics 45(1):255–268. 10.2307/2532051 2720055

[CR25] McBride GB (2005) A proposal for strength-of-agreement criteria for Lin’s concordance correlation coefficient. NIWA Client Report HAM2005-062

[CR26] Neyroud D, Rüttimann J, Mannion AF, Millet GY, Maffiuletti NA, Kayser B et al (2013) Comparison of neuromuscular adjustments associated with sustained isometric contractions of four different muscle groups. J Appl Physiol 114(10):10. 10.1152/japplphysiol.01539.2012 23471948

[CR27] Neyroud D, Maffiuletti NA, Kayser B, Place N (2012) Mechanisms of fatigue and task failure induced by sustained submaximal contractions. Med Sci Sports Exerc 44(7):7. 10.1249/mss.0b013e318245cc4dcc4d. (**PubMed PMID: 22215181**) 22215181

[CR28] Neyroud D, Grosprêtre S, Gondin J, Kayser B, Place N (2018) Test–retest reliability of wide-pulse high-frequency neuromuscular electrical stimulation evoked force. Muscle Nerve 57(1):1. 10.1002/mus.25747 28722822

[CR29] Ochs RM, Smith JL, Edgerton VR (1977) Fatigue characteristics of human gastrocnemius and soleus muscles. Electromyogr Clin Neurophysiol 17(3–4):297–306 923509

[CR30] Place N, Maffiuletti NA, Martin A, Lepers R (2007) Assessment of the reliability of central and peripheral fatigue after sustained maximal voluntary contraction of the quadriceps muscle. Muscle Nerve 35(4):4. 10.1002/mus.20714 17221875

[CR31] Place N, Duclay J, Lepers R, Martin A (2009) Unchanged *H*-reflex during a sustained isometric submaximal plantar flexion performed with an EMG biofeedback. J Electromyogr Kinesiol 19(6):e395–402. 10.1016/j.jelekin.2009.01.001 19216091

[CR32] Place N, Yamada T, Bruton JD, Westerblad H (2010) Muscle fatigue: from observations in humans to underlying mechanisms studied in intact single muscle fibres. Eur J Appl Physiol 110(1):1–15. 10.1007/s00421-010-1480-0 20419312

[CR33] Rodriguez-Falces J, Malanda A, Navallas J (2022) Effects of muscle shortening on single-fiber, motor unit, and compound muscle action potentials. Med Biol Eng Comput 60(2):349–364. 10.1007/s11517-021-02482-z PMC8766404 34936063

[CR34] Rodriguez-Falces J, Place N (2017) Muscle excitability during sustained maximal voluntary contractions by a separate analysis of the M-wave phases. Scand J Med Sci Sports 27(12):1761–75. 10.1111/sms.12819 28028847

[CR35] Rodriguez-Falces J, Place N (2019) Sarcolemmal membrane excitability during repeated intermittent maximal voluntary contractions. Exp Physiol 104(1):136–148. 10.1113/EP087218 30357996

[CR36] Rodriguez-Falces J, Place N (2018) End-of-fiber signals strongly influence the first and second phases of the M wave in the *Vastus lateralis*: implications for the study of muscle excitability. Front Physiol 9:162. 10.3389/fphys.2018.00162 PMC5852100 29568271

[CR37] Rodriguez-Falces J, Maffiuletti NA, Place N (2013) Spatial distribution of motor units recruited during electrical stimulation of the quadriceps muscle versus the femoral nerve. Muscle Nerve 48(5):752–761. 10.1002/mus.23811 24037807

[CR38] Rodriguez-Falces J, Place N (2018) Determinants, analysis and interpretation of the muscle compound action potential (M wave) in humans: implications for the study of muscle fatigue. Eur J Appl Physiol 118(3):3. 10.1007/s00421-017-3788-5 29282530

[CR39] Rodriguez-Falces J, Place N (2014) Effects of muscle fibre shortening on the characteristics of surface motor unit potentials. Med Biol Eng Comput 52(2):95–107. 10.1007/s11517-013-1112-z 24170551

[CR40] Rodriguez-Falces J, Malanda A, Navallas J, Place N (2023) M-wave changes caused by brief voluntary and stimulated isometric contractions. Eur J Appl Physiol 123(9):2087–2098. 10.1007/s00421-023-05228-x PMC10460755 37202629

[CR41] Sale D, Quinlan J, Marsh E, McComas AJ, Belanger AY (1982) Influence of joint position on ankle plantarflexion in humans. J Appl Physiol 52(6):6. 10.1152/jappl.1982.52.6.1636 7107473

[CR42] Smith JL, Martin PG, Gandevia SC, Taylor JL (2007) Sustained contraction at very low forces produces prominent supraspinal fatigue in human elbow flexor muscles. J Appl Physiol 103(2):560–568. 10.1152/japplphysiol.00220.2007 17463302

[CR43] Verges S, Maffiuletti NA, Kerherve H, Decorte N, Wuyam B, Millet GY (2009) Comparison of electrical and magnetic stimulations to assess quadriceps muscle function. J Appl Physiol 106(2):701–710. 10.1152/japplphysiol.01051.2007 18756009

