# Supplementary material for: Reliability of M-wave amplitude measurement from paired stimulations across different muscle groups before and after submaximal contraction to failure
Source: Eur J Appl Physiol. 2026 May 26;126(8):4521–31. doi: 10.1007/s00421-026-06259-w (PMC13668956; doi:10.1007/s00421-026-06259-w)
Supplement: Supplementary file 1 [file 421_2026_6259_MOESM1_ESM.docx]

Table 1. *Number of included participants for knee extensor (KE), plantar flexor (PF), elbow flexor (EF) and thumb adductor (THU) M waves recorded before (Pre) and after (Post) a sustained submaximal contraction sustained to failure.*

|  |  | M100 | |  | M10_1st_ | |  | M10_2nd_ | |  | M_single_ | |
| --- | --- | --- | --- | --- | --- | --- | --- | --- | --- | --- | --- | --- |
|  |  | Pre | Post |  | Pre | Post |  | Pre | Post |  | Pre | Post |
|  |  |  |  |  |  |  |  |  |  |  |  |  |
| KE | VL | 11 | 11 |  | 13 | 13 |  | 13 | 13 |  | 13 | 13 |
|  | VM | 12 | 12 |  | 13 | 13 |  | 13 | 13 |  | 10 | 10 |
|  | RF | 11 | 10 |  | 13 | 13 |  | 13 | 13 |  | 12 | 12 |
|  |  |  |  |  |  |  |  |  |  |  |  |  |
| PF | SOL | 13 | 12 |  | 13 | 13 |  | 13 | 13 |  | 13 | 13 |
|  | GL | 12 | 11 |  | 13 | 13 |  | 13 | 13 |  | 13 | 13 |
|  | GM | 12 | 12 |  | 13 | 13 |  | 13 | 13 |  | 13 | 13 |
|  |  |  |  |  |  |  |  |  |  |  |  |  |
| EF | BRA | 10 | 10 |  | 13 | 13 |  | 13 | 13 |  | 13 | 13 |
|  |  |  |  |  |  |  |  |  |  |  |  |  |
| THU | ADD | 10 | 10 |  | 10 | 10 |  | 10 | 10 |  | 10 | 10 |
|  |  |  |  |  |  |  |  |  |  |  |  |  |

M100, M wave obtained in response to the first (or second for ADD) stimulus of paired stimuli delivered at 100 Hz; M10_1st_, M wave obtained in response to the first stimulus of paired stimuli delivered at 10 Hz; M10_2nd_, M wave obtained in response to the second stimulus of paired stimuli delivered at 10 Hz; M_single_, M wave obtained in response to a single stimulation; VL, *vastus lateralis*; VM, *vastus medialis*; RF, *rectus femoris*; Sol, *soleus*; GL, *gastrocnemius lateralis*; GM, *gastrocnemius medialis*; BRA, *brachioradialis*; ADD, *adductor pollicis*.
